# Aquatic and terrestrial morphotypes of the aquatic invasive plant, *Ludwigia grandiflora*, show distinct morphological and metabolomic responses

**DOI:** 10.1002/ece3.3848

**Published:** 2018-02-04

**Authors:** Kevin Billet, Julien Genitoni, Michel Bozec, David Renault, Dominique Barloy

**Affiliations:** ^1^ ESE, Ecology and Ecosystem Health Agrocampus Ouest, INRA Rennes France; ^2^ UMR CNRS 6553 EcoBio University of Rennes 1 Rennes France; ^3^ Institut Universitaire de France Paris Cedex France

**Keywords:** adaptation, fermentation pathways, glycolysis, hypoxia, water primrose

## Abstract

In the context of expansion of invasive species, survival of invasive plants is conditioned by their ability to adapt. In France, the water primrose *Ludwigia grandiflora*, an aquatic invasive species, invades yet wet meadows, leading to a depreciation of their fodder value. Understanding its potential adaption is necessary to its management, strong differences between both morphotypes were expected. So morphological and metabolic responses to terrestrial environment were analyzed for aquatic and terrestrial morphotypes. All morphological and biomass variables were greater in the terrestrial morphotype than the aquatic morphotype, independent of conditions. In terrestrial condition, both morphotypes showed a high production of sugars in root tissues, especially in the terrestrial morphotype and both morphotypes produced a low level of amino acids in shoot tissues. All results demonstrate that the terrestrial condition seems a stressful situation for both morphotypes, which activates glycolysis and fermentation pathways to improve their survival under hypoxic stress. But, only the terrestrial morphotype has been able to adjust its metabolism and maintain efficient growth. In the future, a differential transcriptomic analysis will be carried out to confirm this result.

## 1 INTRODUCTION

During geographic expansion of invasive plant species, the ability of the organisms to adapt to novel sets of environmental conditions is crucial for survival and establishment success. According to Dietz and Edwards (2006), an invasion can be subdivided into two phases; a primary phase, during which the abundance of a preadapted species increases rapidly (typically in a resource‐rich or disturbed habitat), and a secondary phase, during which spread is contingent upon plastic responses or genetic adaptation to the newly encountered ecological conditions.

The water primrose *Ludwigia grandiflora*, subsp. *hexapetala*, a plant species native to South America, is one of the most important invasive aquatic plants in Europe (Hussner, Windhaus, & Starfinger, 2016). In its native range, *L. grandiflora* is reported in wetlands and in the transition zone between aquatic and terrestrial environments (Thouvenot, Haury, & Thiébaut, 2013). In these habitats, water level fluctuations of up to 3 m during the year occur (Fortney et al., 2004). In its introduced range, it colonizes static or slow‐flowing waters, river banks and wet meadows (Thouvenot et al., 2013). In France, *L. grandiflora* has a strong predominance in the Atlantic region and has invaded many types of fresh water ecosystem such as rivers, channels, ponds, and flooded meadows (Dandelot, Verlaque, Dutartre, & Cazaubon, 2005; Lambert, Dutartre, Coudreuse, & Haury, 2010). As an amphibious plant, it can have different forms as submerged, floating at the water's surface or emergent in terrestrial habitats, rooted in the sediment with floating or emergent shoots/leaves (Hussner et al., 2017). The phylogenetic reconstructions of the *Ludwigia* genus suggest that the aquatic distribution may result from a secondary colonization of these habitats by terrestrial plants (Bedoya & Madriñán, 2015). However, recently, specimens of *L. grandiflora* have propagated in wet meadows in France (Haury et al., 2011). As 5–10 years are necessary for the invasion of meadows, the transition from aquatic habitat to the terrestrial habitat is not spontaneous and requires an adaptation time for *L. grandiflora*. So the quasi absence of *L. grandiflora* into terrestrial some few years ago is clearly intriguing.

Several localities of the French Atlantic coast are now being invaded by terrestrial form, where it is continuously expanding with more than 1300 ha in 2017 (Haury, unpublished data). For instance, in the national Park of Brière (nearby Saint Nazaire, Loire Atlantique, France), the terrestrial areas colonized by *L. grandiflora* were multiplied by five between 2011 (156 ha) and 2014 (848 ha), leading to a depreciation of the fodder value of meadows and resulting in the abandonment of pasturing (Haury, Damien, Maisonneuve, & Bottner, 2012). The invasion of terrestrial habitat by *L. grandiflora* can be considered as a secondary invasion following the primary aquatic invasion (primary habitat) of French Atlantic coast.

Aquatic and terrestrial environments differ greatly in terms of oxygen availability and have likely contributed shaping the aquatic and terrestrial morphological types (morphotypes) of *L. grandiflora*. The terrestrial morphotype is characterized by a bushy morphology, with shorter internodes and stems, as well as more secondary ramifications. Conversely, in aquatic habitats, the plant architecture is simpler (Haury et al., 2014) and is most probably driven by the limited gas exchanges of water submerged leaves as O_2_ and CO_2_ have 10,000‐fold lower diffusion coefficients in water compared to air (Voesenek, Colmer, Pierik, Millenaar, & Peeters, 2007). For completely submerged plants, like macrophytes, direct access to atmospheric O_2_ is excluded and may cause anoxic or hypoxic conditions. Their only source of oxygen is produced during photosynthesis and diluted in water (Sand‐Jensen, Pedersen, Binzer, & Borum, 2005). In these conditions, enhanced glycolytic fluxes and ethanolic fermentation are often characterizing the metabolism of aquatic plants (Fukao & Bailey‐Serres, 2004), as the faster consumption of glucose to maintain ATP production (van Dongen et al., 2011; Narsai, Rocha, Geigenberger, Whelan, & van Dongen, 2011). In addition to the accumulation of ethanol and lactate during ethanolic fermentation, increased amounts of alanine, succinate, and γ‐aminobutyric acid (GABA) are often measured in aquatic plants (Antonio et al., 2016).

In order to survive prolonged hypoxic conditions, aquatic plants have also developed adaptive mechanisms at the structural level. For roots, the commonest morphological and anatomical changes include the formation of adventitious roots, aerenchyma, and hypertrophied lenticels. These modifications improve the capacity for oxygen capture and transportation to submerged tissues in aquatic, semi‐aquatic, and terrestrial plants (Gladish, Xu, & Niki, 2006; Gunawardena, Pierce, Jackson, Hawes, & Evans, 2001; Justin & Armstrong, 1987; Malik, Colmer, Lambers, & Schortemeyer, 2003). Similar to most aquatic plant species, two types of roots are observed in *L. grandiflora* (i) roots that adsorb nutrients and attach the plant to the soil, and (ii) adventitious roots, which are located along the stems and ensure oxygen uptake (EPPO, 2011). Semi‐aquatic and aquatic species developed a large amount of aerenchyma in roots, but also in stems and leaves (Jung, Lee, & Choi, 2008; Nowak, Ono, & Cronk, 2010).

The water primerose is a heterophyllous aquatic plant that produces different types of leaves below and above water during its development (EPPO, 2011). Wells and Pigliucci (2000) showed earlier that in amphibious species, submerged leaves are thin and lack both a cuticle and stomata, whereas aerial leaves are thicker, cutinized, and bear stomata. When aerial leaves of amphibious species become submerged, CO_2_ entry occurs predominantly via diffusion across the cuticle (Frost‐Christensen & Floto, 2007). Similar characteristics may occur in *L. grandiflora*, but this has never been examined before.

In this work, we compared the performance of the terrestrial and aquatic morphotypes of *L. grandiflora* to understand the effect of the habitat change. We expected strong morphological and physiological differences between both morphotypes. We specifically aim (i) to investigate the differences and similarities in plant development and primary metabolism responses of both morphotypes in their original condition and (ii) to compare the same variables in a reversed condition (aquatic condition for terrestrial morphotype and terrestrial condition for aquatic morphotype).

We focused our study on a population of *L. grandiflora* that appeared in 1995 in its aquatic form. In 2000, this morphotype invaded meadows, leading to appearance of the terrestrial morphotype. We collected *L. grandiflora* plants from terrestrial and aquatic habitats and grew them either in their original sampling conditions or reversed their original ecological situation. Terrestrial morphotype comes from the aquatic population and as *L. grandiflora* is a vegetative reproduction species, we consider that terrestrial and aquatic morphotypes correspond to a same genotype.

## 2 MATERIALS AND METHODS

### 2.1 Collection of the plants and experimental design

Aquatic (Am) and terrestrial (Tm) morphotypes of *L. grandiflora* were collected in autumn of 2014 from swamps of Mazerolles (nearby Nantes, France, N47 23.260, W1 28.206). They were grown under controlled conditions at 22°C and a 16‐hr/8‐hr (light/dark) cycle.

Starting from the apex, a 10‐cm portion of the stem was collected, without roots, buds, and lateral stems. Then, a preconditioning period of 2 weeks was applied to promote the development of roots in different aquariums, as described in Figure S1. During the first week, both aquatic and terrestrial plants were developed in the aquatic condition, with a total immersion of the stems. Then, plants from both morphotypes were allocated to one of two experimental conditions for one week: (i) terrestrial condition: plants were grown in a mixture of ^1^/_3_ soil, ^1^/_3_ sand, ^1^/_3_ loam where the level of water was maintained flush, hereafter referred to as Am‐t and Tm‐t for aquatic and terrestrial morphotypes, respectively, and (ii) aquatic condition: plants were placed in the same substrate and were submerged into tap water—these plants are referred to as Am‐a and Tm‐a for aquatic and terrestrial morphotypes, respectively.

After preconditioning, aquatic and terrestrial morphotypes were randomly placed in containers (Length × Width × Height: 8 cm × 8 cm × 15 cm) in aquatic condition for Am‐a and Tm‐a and terrestrial condition for Am‐t and Tm‐t, under controlled conditions (22°C, 16‐hr day/8‐hr night; Figure S1).

Measurements of morphological traits and metabolomic patterns of the plants were conducted 1 week after this conditioning period; two and three biological replicates were carried out for the morphological and metabolomic analyses, respectively.

### 2.2 Morphological analyses

For each experimental treatment, corresponding to aquatic and terrestrial morphotypes grown into two experimental conditions, eight plants were analyzed. The following morphological and biological traits were measured:

1. Length of the plant (LP), number of internodes (nbI), and number of leaves (nbL), which characterized the morphology of the plant;
2. Number of nodes with roots (nbNR), number of nodes with leaves and roots (nbNLR), and number of nodes with buds (nbNB); these three variables were associated with the fitness of the plant. In this work, fitness of *L. grandiflora* was considered to be the ability to produce propagules from buds or nodes with leaves and/or roots. Indeed, *L. grandiflora* shows a high regeneration capacity and the ability to form new shoots from single nodes (with or without leaves) from a small (>1 cm) plant fragments (Hussner, 2009).
3. Biomass of the plants was determined by measuring the fresh mass of shoots (FMS) and of roots (FMR). Their respective dry masses (DMS and DMR) were determined after oven drying the samples at 105°C for 48 hr. The ratios of fresh/dry mass of shoots (Sr) and roots (Rr), and ratio of ratios (rr = Sr/Rr) were calculated to determine water contents.

### 2.3 Metabolomic fingerprint

For each experimental treatment, six plants were randomly collected and pooled to constitute one sample. To standardize the samples, ten centimeters from the top of the stem (including stem, leaves, and buds) and roots were sampled and immediately snap‐frozen in liquid nitrogen. Then, samples were stored at −80°C until being ground into liquid nitrogen. Samples were lyophilized over 48 hr using a Cosmos 20K (Cryotec, Saint‐Gély‐du‐Fesc, France). Gas chromatography coupled with mass spectrometry (GC‐MS) was used to scan for the 60 metabolites in our database, encompassing amino acids, organic acids, polyamines, polyols, and sugars. We used the method described in Serra et al. (2013). Briefly, for each sample, an aliquot of 10 mg of lyophilized powder was homogenized in 600 μl of a solution of ice‐cold methanol/chloroform (2:1, v/v). Then, a volume of 400 μl of ultra‐pure water was added. Samples were homogenized and centrifuged for 10 min at 4,000 ***g*** (4°C). One hundred and twenty microliters of the upper phase containing metabolites was transferred to new glass vials. Samples were vacuum dried (Speed Vac Concentrator, MiVac, Genevac Ltd., Ipswich, England). The derivatization of the samples was conducted with a CTC CombiPAL autosampler (CTC Analytics AG, Zwingen, Switzerland), and with the GC‐MS settings that were described by Serra et al. (2013). Chromatograms were analyzed with XCalibur 2.0.7. Concentration of each metabolite was calculated using individual quadratic calibration curves.

### 2.4 Statistical analysis

The effect of the experimental treatment on the morphological and metabolic phenotypes of the plants was assessed by running principal component analyses (PCAs). Analyses of variance (ANOVAs) was conducted to assess the effect of the experimental conditions on the morphological traits of the plants and metabolite concentrations, with morphotype, experimental conditions, and replicates as factors, and their two‐way interactions. The residuals normality and the homoscedasticity were verified by Shapiro–Wilk's and Bartlett's tests, and normality was achieved by transforming morphological traits and the metabolite concentrations (Cos or Log_10_; Table S1). The Tukey honestly significant difference test was used to compare means for morphological and metabolomic data, respectively. Statistical analyses were performed with the software R 3.2.2 (R Development Core Team 2008) using the package and plugin FactoMineR (Lê, Josse, & Husson, 2008) and the interface Rcmdr (Fox, 2005). Finally, we tried to link metabolites whose concentration varied among the experimental treatments with metabolic pathways. To achieve this goal, we performed pathway enrichment analyses in Metaboanalyst 3.0 (Xia, Sinelnikov, Han, & Wishart, 2015). Fisher's exact test algorithm was performed for these pathway analyses, with *Arabidopsis thaliana* as the reference model. In this procedure, the number of hits between the metabolites in our dataset and all metabolites of a given pathway were calculated. Data presented in the figures are untransformed.

## 3 RESULTS

### 3.1 Morphology

**Figure 1:**
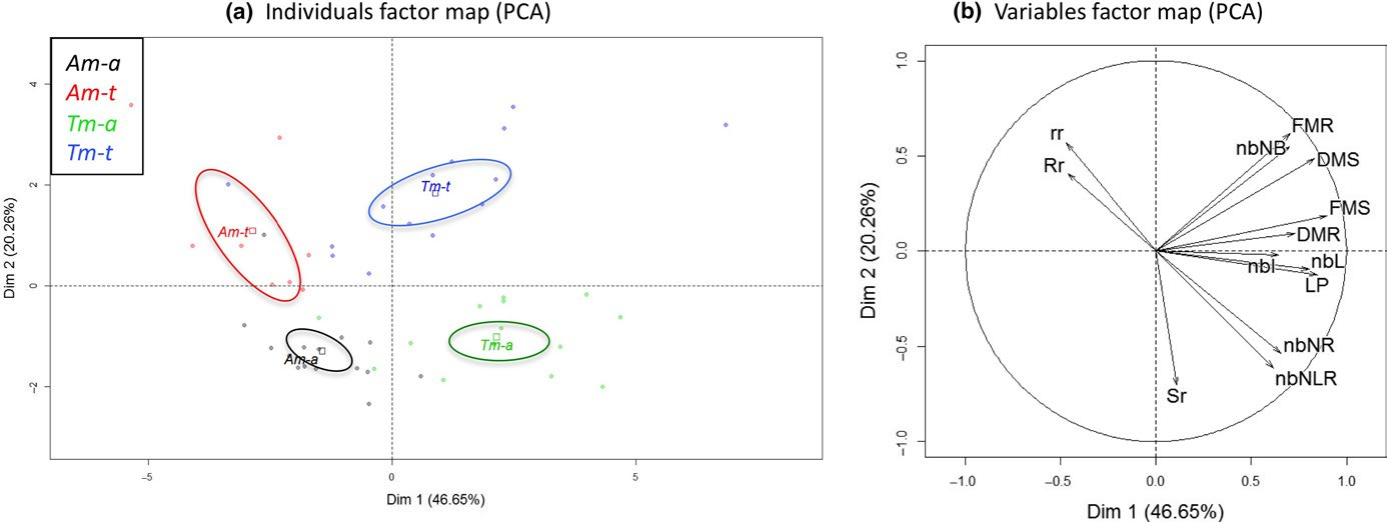
Principal component analysis (PCA) of morphological traits for the aquatic and terrestrial morphotypes conducted in aquatic (Am‐a, Tm‐a) or terrestrial (Am‐t, Tm‐t) conditions. (a) Individuals factor map. Each data point represented one plant. (b) Variables factor map. Variables were the length of the plant (LP); the number of internodes (nbI); the number of leaves (nbL); the number of nodes with roots (nbNR) and with leaves and roots (nbNLR); the number of nodes with buds alone (nbNB) and with buds and leaves (nbNBL) and/or roots (nbNBLR and nbNBR); the fresh mass of stems (FMS) and roots (FMR); dry mass of stems (DMS) and roots (DMR); ratios of fresh/dry mass for stems (Sr) and for roots (Rr); and the ratio of ratios (rr = Sr/Rr)

The separation of the groups according to the experimental treatments is presented in Figure 1. The first axis of the PCA explained 46.65% of the total inertia and separated the Am morphotype of *L. grandiflora* from the Tm morphotype. On this axis, the seven variables being correlated at least at 70% were conserved for investigating the main differentiating factors between morphotypes (Figure 1b). Tm was characterized by greater plant length, fresh and dry masses of shoots and roots, and number of nodes with buds (Figures 1b and S2). The second axis of the PCA explained 20.26% of the total inertia, and separated plants maintained under aquatic and terrestrial conditions (Figure 1a). In the aquatic condition, *L. grandiflora* plants showed a greater number of nodes with leaves and roots, a larger shoots ratio of fresh and dry masses, and a smaller fresh mass of roots as compared to plants from terrestrial condition (*r* ^2^ > 60%; Figures 1b and S2).

**Figure 2:**
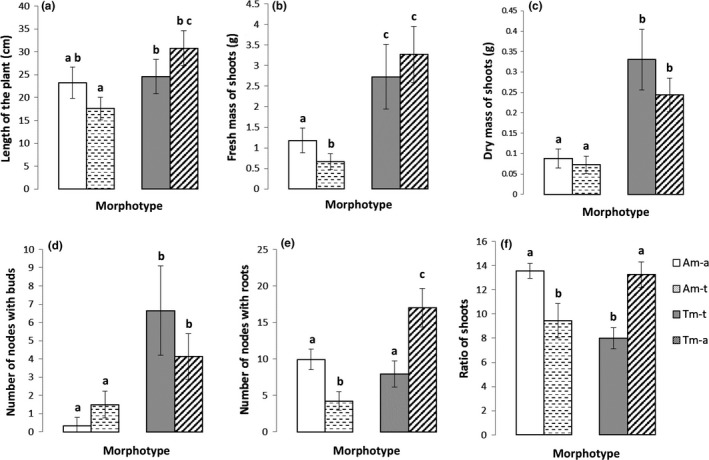
Variation of different traits of *Ludwigia grandiflora* for the aquatic and terrestrial morphotypes grown in aquatic (Am‐a, Tm‐a) or in terrestrial (Am‐t, Tm‐t) conditions. (a) the length of the plant (LP), (b) the fresh mass of shoots (FMS), (c) the dry mass of shoots (DMS), (d) the number of nodes with buds (nbNB), (e) the number of nodes with roots (nbNR), and (f) the ratio of shoots (Sr). Same letter indicates non‐different values (α = .05)

Morphotypes and experimental conditions significantly affected the studied variables (Table 1). All morphological, biomass and fitness variables were significantly greater in the terrestrial morphotype (Tm) than in the aquatic morphotype (Am; *p* values ranging from <.01 to <.001; Table 1, Figure 2). Even in aquatic condition, assumed to be less favorable for the terrestrial morphotype (Tm‐a), terrestrial morphotype presented length values of about 30.8 cm (LP; Figure 2a), fresh or dry mass values of shoots (FMS, DMS; Figure 2b,c) of about 3.27 g and 0.24 g, respectively, significantly more than those of the aquatic morphotype (Am‐a (LP) = 23.2 cm, Am‐a (FMS) = 1.18 g, Am‐a (DMS) = 0.09 g). A similar pattern was observed for fitness variables: Tm‐a produced 8 and 1.5 times more buds (Figure 2d) and nodes with roots (Figure 2e), respectively, than Am‐a. Am showed significantly greater water content in shoots or roots than in Tm (*p* value < .01 and *p* value = .016, respectively; Table 1).

**Table 1:** Variations of morphological and biomass variables, and variables associated with the fitness and water content as a function of morphotype or condition

	Morphological trait (code)	Morphotype				Condition			
		Inequality	*df*	*F*	*p*	Inequality	*df*	*F*	*p*
Morphological variables	Length of the plant (LP)	**Am < Tm**	1	12.57	***	*a > t*	1	10.54	**
	Number of internodes (nbI)	**Am < Tm**	1	21.13	***				NS
	Number of leaves (nbL)	**Am < Tm**	1	251.6	***	*a > t*	1	91.38	*
Biomass variables	Fresh mass of shoots (FMS)	**Am < Tm**	1	65.59	***	*a > t*	1	6.749	*
	Fresh mass of roots (FMR)	**Am < Tm**	1	79.89	***	**a < t**	1	10.84	**
	Dry mass of shoots (DMS)	**Am < Tm**	1	101.2	***				NS
	Dry mass of roots (DMR)	**Am < Tm**	1	88.59	***				NS
Variables associated with the fitness	Number of nodes with roots (nbNR)	**Am < Tm**	1	20.2	***	*a > t*	1	52.82	***
	Number of nodes with leaves and roots (NbNLR)	**Am < Tm**	1	7.724	**	*a > t*	1	71.92	***
	Number of buds (NbB)	**Am < Tm**	1	33.06	***	**a < t**	1	5.74	*
Water content	Shoots ratio (Sr)	*Am > Tm*	1	9.052	**	*a > t*	1	284	***
	Roots ratio (Rr)	*Am > Tm*	1	6.269	*	**a < t**	1	7.588	**
	Ratio of ratios (rr)				NS	**a < t**	1	28.2	***

Principal morphological traits significantly higher in the terrestrial morphotype (Tm) than in the aquatic morphotype (Am) are represented in bold, and in italic when Am is significantly higher than Tm. When the terrestrial condition (−t) values are greater than aquatic (−a), it is represented in bold, in italic when (−a) was significantly greater than (−t). Significance codes: 0 “***”; .001 “**”; .01 “*”; nonsignificant “NS.”

Independent of the morphotype, most of morphological and biomass variables were enhanced significantly in plants maintained under the aquatic condition, except for the number of buds and the water content of roots (*p* value < .01 to *p* value < .0001; Table 1).

In the terrestrial condition, both morphotypes had less water content in shoots than in aquatic condition. The ratio of shoots (Sr) was significantly different in aquatic or terrestrial conditions (Figure 2f). However, changing conditions did not disturb significantly the water content (rr) in aquatic and terrestrial morphotypes (Table 1).

### 3.2 Metabolism

#### 3.2.1 Metabolomic analyses of shoot tissues

**Figure 3:**
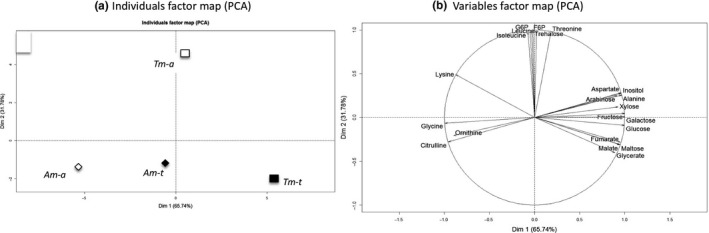
Principal component analysis (PCA) of shoot metabolite profiles for the aquatic and terrestrial morphotypes (Am, Tm) conducted in aquatic (Am‐a, Tm‐a) or in terrestrial (Am‐t, Tm‐t) conditions. (a) Individuals factor map. Each data point represented the mean of the metabolite in the three biological repetitions. (b) Variables factor map

**Figure 4:**
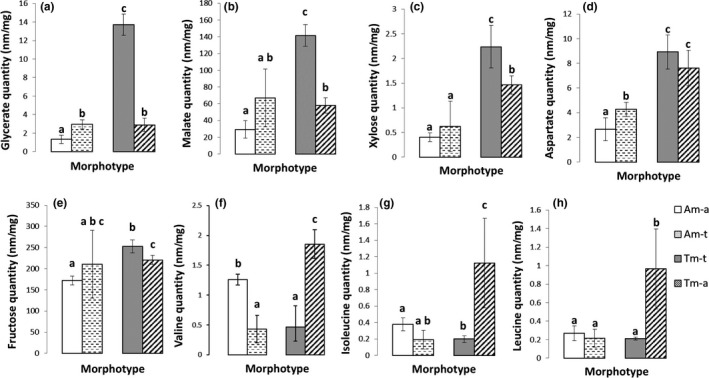
Changes in glycerate (a), malate (b), xylose (c), aspartate (d), fructose (e), valine (f), isoleucine (g), and leucine (h) in *Ludwigia grandiflora* shoots of aquatic and terrestrial morphotypes in aquatic (Am‐a, Tm‐a) or in terrestrial (Am‐t, Tm‐t) conditions. All values were expressed in nmol/mg of dry mass of stems. Same letter indicates non‐different values (α=0.05).

Among the 47 metabolites detected in shoot tissues, 22 compounds were at least 75% correlated with axes of the PCAs and were kept to perform this statistical procedure. The first axis of the PCA accounted for 65.74% of the total inertia. It separated Am and Tm, with Am‐a and Tm‐t being clearly opposed (Figure 3a). Plants from the Am‐a treatment were characterized by higher levels of citrulline, glycine, lysine, and ornithine in the shoots (Figures 3b and S3). Plants from the Tm‐t treatment exhibited higher amounts of arabinose, fructose, fumarate, galactose, glucose, malate, maltose, and xylose (Figures 4 and S3).

After this descriptive analysis, ANOVA analyses were run. In the terrestrial condition, shoots of Tm‐t contained more organic acids than shoots from Am‐t (confidence interval, α = .05). It concerned the glycerate (ratio_(Tm/Am)_ = 4.65, Figure 4a), the malate (ratio_(Tm/Am)_ = 2.11; Figure 4b), and the xylose (ratio_(Tm/Am)_ = 3.6, Figure 4c). In the aquatic condition, the shoots of Tm‐a had greater contents of 16 of the 22 detected metabolites than the shoots of Am‐a (confidence interval, α = .05). It concerned organic acids as the glycerate (ratio_(Tm/Am)_ = 2.19, Figure 4a), the malate (ratio_(Tm/Am)_ = 1.98, Figure 4b) and the xylose (ratio_(Tm/Am)_ = 3.64, Figure 4c) as well as most of amino acids as the aspartate (ratio_(Tm/Am)_ = 2.86, Figure 4d).

The second axis of the PCA accounted for 31.78% of the total inertia, and a clear‐cut separation appeared in between Tm‐a and the three others groups. Tm‐t plants had greater amounts of fructose 6‐phosphate (F6P), glucose 6‐phosphate (G6P), isoleucine, leucine, trehalose, and threonine (Figures 3a,b and 4). ANOVA analyses revealed that valine, isoleucine, and leucine had lower concentrations (*p* value < .003, Figure 4f–h) in Am independently of condition (Am‐a or Am‐t), and also in Tm‐t, than in Tm‐a.

**Figure 5:**
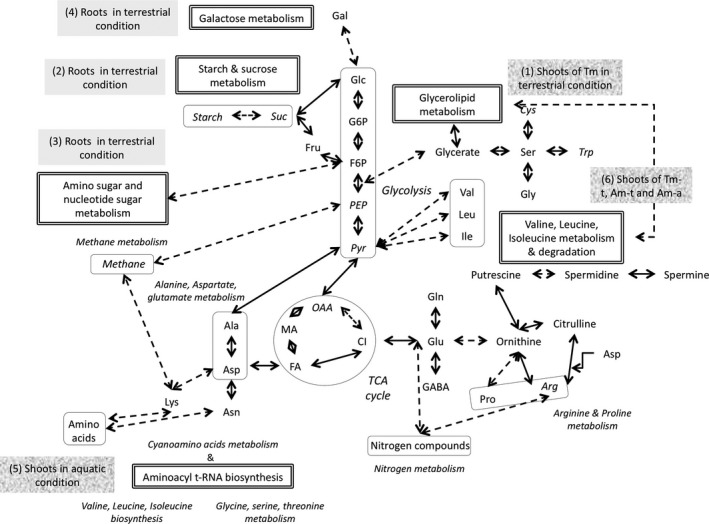
Overview of the metabolism of sugars, amino acids, and related compounds. (Elaborated from kegg pathways; http://www.kegg.jp.) The metabolites analyzed by mass spectrometry (GC‐MS) and metabolomic pathways identified by MetaboAnalyst's server were indicated in a character box. Metabolites and others pathways noted in italic were not screened or not identified by MetaboAnalyst's server. The adaptation of *Ludwigia grandiflora* to terrestrial condition mobilized four metabolomic pathways (numbers 1–4) in roots or shoots. The aminoacyl t‐RNA biosynthesis pathway (no. 5) was specifically involved in shoots in aquatic condition. The valine, leucine, isoleucine metabolism, and degradation (no. 6) were only not involved in terrestrial morphotype in the terrestrial condition (Tm‐a, Terrestrial morphotype in aquatic condition; Am‐a and Am‐t, Aquatic morphotype in aquatic and terrestrial conditions, respectively). Ala, Alanine; Arg, Arginine; Asn, Asparagine; Asp, Aspartate; Cys, Cysteine; Ci, Citrate; FA, Fumarate; Fru, Fructose; F6P, Fructose 6‐phosphate; Gln, Glutamine; Glu, Glutamate; Gly, Glycine; Ile, Isoleucine; GABA, Gamma aminobutyric acid; Gal, Galactose; Glc, Glucose; G6P, Glucose 6‐phosphate; Leu, Leucine; Lys, Lysine; MA, Malate; OAA, Oxaloacetate; PEP, Phosphoenolpyruvic acid; Pro, Proline; Pyr, Pyruvate; Ser, Serine; Suc, Sucrose; TCA cycle, Tricarboxylic acid cycle; Trp, Tryptophan; and Val, Valine

According to metabolite variations, the activity of three principal metabolic pathways was altered in shoots of *L. grandiflora* (Figure 5): the pathway of the glycerolipid metabolism (Holm *p* value < .01), the aminoacyl‐tRNA biosynthesis pathway (Holm *p* value = .0654), and the valine, leucine, and isoleucine degradation (Holm *p* value = .0654).

#### 3.2.2 Metabolomic analysis of root tissues

**Figure 6:**
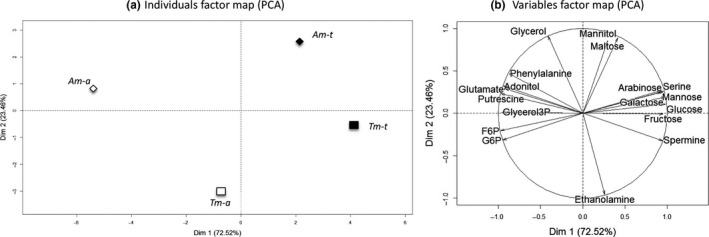
Principal component analysis (PCA) of root metabolite profiles for the aquatic and terrestrial morphotypes conducted in aquatic (Am‐a, Tm‐a) or in terrestrial (Am‐t, Tm‐t) conditions. (a) Individuals factor map. Each data point represented the mean of the metabolite in the three biological repetitions. (b) Variables factor map

Eighteen of the 47 quantified metabolites were used for running the PCA (*R* ^2^ > 80%). The first axis explained 72.52% of the total inertia, and discriminated plants from aquatic and terrestrial conditions (Figure 6a), with Am‐a and Tm‐t again being clearly opposed. Am‐a was characterized by higher amounts of adonitol, F6P, G6P, glutamate, glycerol 3‐phosphate, phenylalanine, and putrescine in roots (*R* ^2^ > 84%; Figures 6b and S4). Conversely, Tm‐t had higher levels of arabinose, fructose, galactose, glucose, mannose, serine, and spermine (*R* ^2^ > 87%; Figures 6b and S4). The second axis distinguished Am‐t from Tm‐a morphotypes, according to higher contents in glycerol, mannitol and maltose in Am‐t. Conversely, Tm‐a plants had more amounts in ethanolamine (*R* ^2^ > 92%). These two axes explained 95.98% of the total inertia and resulted in a clear‐cut separation of the groups, each being projected in one part of the PCA plane.

**Figure 7:**
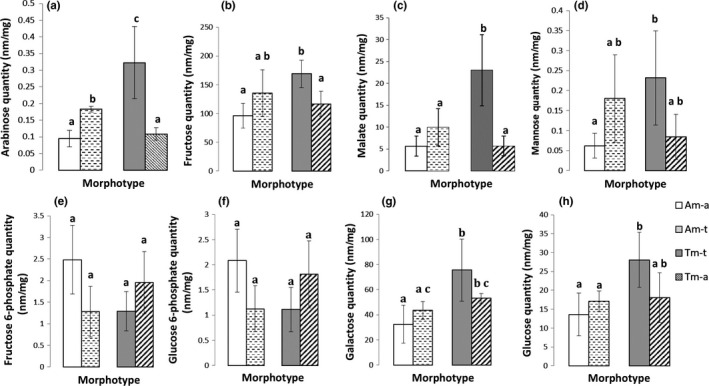
Changes in arabinose (a), fructose (b), malate (c), mannose (d), frutose 6‐phosphate (e), glucose 6‐Phosphate (f), galactose (g), and glucose (h) in *Ludwigia grandiflora* roots of aquatic and terrestrial morphotypes in aquatic (Am‐a, Tm‐a) or terrestrial (Am‐t, Tm‐t) conditions. All values were expressed in nmol/mg of dry mass of roots. Same letter indicates non‐different values (α = .05)

All sugars quantified from the root tissues were in lower concentrations in Am‐a plants as compared to Tm‐t plants (Figure 7). In the terrestrial condition, independent of morphotype, lesser amounts of F6P (ratio_(t/a)_ = 0.58; *p* value < .01) and G6P (ratio_(t/a)_ = 0.57; *p* value < .01), and greater quantities of arabinose (ratio_(t/a)_ = 2.51; *p* value < .05; Figure 7a), fructose (ratio_(t/a)_ = 1.43; *p* value < .05; Figure 7b), malate (ratio_(t/a)_ = 2.91; *p* value < .01; Figure 7c), and mannose (ratio_(t/a)_ = 2.81; *p* value < .01; Figure 7d) were found in the roots of *L. grandiflora*. The roots of Tm‐a had similar values as Am‐a in fructose (Figure 7b), galactose, glucose, and mannose (Figure 7d,e).

According to metabolite variations, the activity of four metabolic pathways was altered: metabolism of amino sugar and nucleotide sugar (Holm *p* value < .01), galactose (Holm *p* value < .01), fructose and mannose (Holm *p* value < .01), and the starch and sucrose (Holm *p* value < .05). These pathways seemed to be upregulated in roots of Tm‐t, and, to a lesser extent, in aquatic Am‐t.

## 4 DISCUSSION

Using a large range of morphological measurements, we demonstrated that the terrestrial morphotype (Tm) outcompeted its aquatic counterpart in both terrestrial and aquatic habitats. The terrestrial morphotype produced plants of higher biomasses and had higher numbers of leaves and roots. While the terrestrial morphotype could be described a fast‐growing plant, whereas aquatic morphotype is characterized by comparatively low growth performance. We suggest that this distinction may be partially supported by an increased root network in Tm, especially in the terrestrial condition, as it has been described under natural conditions by Haury et al. (2014). In addition, if the capacity for CO_2_ uptake is required for achieving a higher relative growth of the Tm, this morphological change is not sufficient for supporting its higher growth rate, which also involves a higher efficiency in acquiring and assimilating available nutrients.

### 4.1 Biomass allocation of *L. grandiflora* depends of the aquatic or terrestrial habitats

Biomass allocation reveals the survival strategies and adaptations to the environmental of the (micro)habitats where plants are developing (Xie, An, & Wu, 2005). Several invasive aquatic weeds are known for their ability to respond to different water levels by adjustments of the root system and/or biomass allocation (Geng et al., 2006; Hussner & Meyer, 2009; Hussner, Meyer, & Busch, 2009; Kercher & Zedler, 2004; Li, Yu, & Xu, 2011). All results indicated that *L. grandiflora* was well adapted to the terrestrial habitat because of its biomass allocation, morphological strategies that depended on the increase in root biomass allocation and water content. Significant morphological changes were also found in *L. grandiflora* plants from aquatic conditions, which developed a larger fresh mass of shoots as compared to plants from the terrestrial condition. Conversely, a larger fresh mass of roots was measured in *L. grandiflora* plants from the terrestrial condition. Accordingly, Hussner (2010) showed that the strong abilities of *L. grandiflora* to grow on drained soils were supported by an increase in the relative amount of root biomass and by changes in the development of the root system. Haury et al. (2014) also observed that the biomasses of field‐collected *L. grandiflora* from meadows were characterized by twofold increase than in dykes and the percentage of roots was slightly higher in plants from meadows than in those from dykes.

### 4.2 Colonization of terrestrial habitats is supported by metabolic adjustments

When released into a new geographic area, the habitats that best match with the biological requirements of the species should be colonized first, and the invasion success depends on propagules’ pressure (Colautti, Grigorovich, & MacIsaac, 2006). Specimens of *L. grandiflora* first settled in different types of ponds and marshes (Lambert et al., 2010) suggesting that this preferred habitat should maximize the biological performance of the plants as compared to their relatives thriving in supposedly less favorable terrestrial habitats. In this work, we thus assumed that the colonization of terrestrial zones corresponded to secondary habitats for this invasive plant species. However, we did not verify this assumption and found that the terrestrial morphotype had higher biological performance, whatever the experimental situation. In order to better understand the changes that facilitated the expansion to terrestrial habitats, we described and compared the functional responses of the two morphotypes throughout metabolomic experiments.

In the terrestrial condition, the amounts of the branched amino acids (BCAAs) isoleucine, leucine, and valine were reduced in the stems of both morphotypes. These three amino acids share four common enzymes in their biosynthesis pathways and are thus jointly regulated (Joshi, Joung, Fei, & Jander, 2010). Protein degradation may have contributed to the elevation of the levels of these three amino acids. BCAAs can indeed provide alternative carbon sources for plants during stressful conditions (Taylor, Heazlewood, Day, & Millar, 2004), and the complete oxidation of these amino acids might have fuelled the particular needs of plants growing in supposedly stressful terrestrial condition. Finally, several amino acids, including BCAAs, play important roles in stressed plants through their osmoprotective action (Campalans, Messeguer, Goday, & Pages, 1999).

Irrespective of morphotypes, three sugar metabolic pathways were enhanced in the root system of *L. grandiflora* grown in the terrestrial condition (starch and sugar metabolism, galactose metabolism, and amino sugar and nucleotide sugar metabolic pathways). In organs of wetland plant species, reserves of carbohydrates and ethanol fermentation are equally important in maintaining glycolysis and energetic metabolism in general (reviewed in Nakamura et al., 2012). In our study, the significant decrease in glucose‐6‐phosphate and fructose‐6‐phosphate levels measured in both morphotypes, and the concomitant increase in fructose and mannose concentrations pinpointed the enhanced glycolytic activities and energetic needs of plants developing in terrestrial condition. The major fermentation end products of ethanol fermentation are lactate and pyruvate, and deficiency of oxygen is also associated with elevation of alanine, GABA, succinate, and occasionally malate (Dixon, Hill, Jackson, Ratcliffe, & Sweetlove, 2006; Drew, 1997). As quantities of malate were greater in plants grown in the terrestrial condition than in those grown in the aquatic condition, it is possible that the former may have partially relied on ethanol fermentation for energy production in their root system. Fermentation processes drastically increase the demand for carbohydrates, and sugars are thus crucially important for surviving prolonged hypoxic conditions (Nakamura et al., 2012). Our results suggested that both morphotypes were able to active glycolysis and fermentation pathways in root tissues in response to the terrestrial condition. However, if glycolysis and fermentation pathways seemed to be similarly regulated between both morphotypes, root system were more developed in Tm. Poorter and Nagel (2000) reported that plants increase biomass allocation to roots in the presence of low levels of belowground resources, such as water and nutrients. This trait could correspond to a root adaptive response of *L. grandiflora* in a terrestrial environment.

### 4.3 Adaptation to terrestrial habitats launched a new morphotype with new ability to endure stressful conditions

The greater content of glycerate in Tm‐t than those observed for Am‐t, Tm‐a, and Am‐a, suggests that Tm‐t plants may have enhanced production of glycerolipids in shoot tissues. The decreased amounts of valine, leucine, and isoleucine from Tm‐t plants support this assumption and may evidence the increased activity of the glycerolipid metabolism pathway (Figure 5; Hildebrandt, Nesi, Araujo, & Braun, 2015). Glycerolipids are the main components of biological membranes, especially photosynthetic membranes, like in thylakoids (Boudière et al., 2014), whose amounts might have been increased in Tm‐t, in parallel to the general increase of all morphological and biomass variables. In addition, glycerolipids are also one constituent of the cuticular that coats the surface of plants, providing the crucial hydrophobic barrier that prevents water loss (Bird, 2008).

In addition to glycerate, Tm‐t plants also had more malate amounts than the three other morphotypes. *Ludwigia grandiflora* is described as a C4 plant (Madanes, Quintana, Kandus, & Bo, 2015). In these plants, C4 pathway requires the close integration of distinct photosynthetic processes: phosphoenolpyruvate (PEP) carboxylation and regeneration through the Calvin cycle (Gowik & Westhoff, 2011). For C4 plants, CO_2_ is converted to bicarbonate by carbonic anhydrase, and fixed by phosphoenolpyruvate carboxylase (PEPC) using PEP as CO_2_ acceptor. The resulting oxaloacetate is rapidly converted to the more stable C4 acids malate whose amounts are high in Tm‐t plants, or aspartate. These acids are then decarboxylated to supply CO_2_ for ribulose bisphosphate carboxylase oxygenase (RuBisCo). The product of this reaction is 3‐phosphoglycerate (glycerate‐3‐phosphate).

### 4.4 Terrestrial morphotype surprisingly outcompetes aquatic morphotype when reared under the aquatic condition

Am‐a and Tm‐a have higher contents of glycine and lysine, and, for Tm‐a, higher levels of isoleucine, leucine, and threonine, in shoot tissues than plants from the terrestrial condition (Am‐t and/or Tm‐t). Our analysis of metabolic pathways revealed that the aminoacyl‐tRNA biosynthesis pathway was promoted for both morphotypes grown in the aquatic condition (Figure 5). Aminoacyl‐tRNAs are key components of protein synthesis and consist of amino acids esterified to the 3′‐end of tRNA, which are essential for ribosomal protein synthesis (Ibba, Curnow, & Söll, 1997). As the terrestrial morphotype of *L. grandiflora* showed a greater amount of isoleucine, leucine, and threonine, especially in the shoots in the aquatic condition, we suggest that this morphotype should be particularly efficient at using the aminoacetyl‐tRNA biosynthesis for the production of these amino acids.

Amino acids are essential precursors for a large array of metabolites with multiple functions in plant growth and environmental stress responses (Less & Galili, 2008), and amino acids can also have direct protective mechanism in many terrestrial plants (Rhodes, Verslues, & Sharp, 1999). The terrestrial morphotype of *L. grandiflora* could experience the aquatic condition as a stress and could accumulate amino acids to protect the plant cells from damage (Rhodes et al., 1999). This hypothesis is supported by the fact that the Am‐a seemed able to catabolize these three amino acids (Figure 5).

In stressful conditions, increased amino acid concentrations have been associated with decreased growth rates in terrestrial and aquatic plants (Maggio et al., 2002; Wallace, Secor, & Schrader, 1984). In the aquatic condition, most of morphological and biomass variables observed were higher in Tm than in Am suggesting that amino acid accumulation was not associated to reduced growth of Tm. Furthermore, as observed in terrestrial condition, Tm produced higher malate and pyruvate quantities than those observed for Am. This suggests that Tm could have also a high C4 photosynthesis activity in the aquatic condition as it was observed in terrestrial condition. Involvement of both metabolic pathways could reflect an adaptive response of terrestrial morphotype to the aquatic condition.

## 5 CONCLUSION

In this work, we have demonstrated that the terrestrial morphotype of *L. grandiflora* showed higher growth capacities in both terrestrial and aquatic habitats as compared to the aquatic morphotype confirming our hypothesis. When transferred to terrestrial conditions, both morphotypes activated glycolysis and fermentation pathways, but, only the terrestrial morphotype had the capacity to maintain efficient growth. In the future studies, comparative analysis of both morphotypes could be carried out to determine whether the terrestrial morphotype shows a loss of secondary tissues and formation of aerenchyma in anoxic conditions, as reported in helophytes. To go further in the understanding of adaptive mechanisms of *L. grandiflora* to terrestrial condition, omics analysis will be realized.

Finally, we suggest focusing management actions on the terrestrial morphotype in order to control the expansion of *L. grandiflora*. Recent studies that assessed the effects of climate changes on the potential distribution of *L. grandiflora* predicts future increase in the size of its bioclimatic range, especially into Ireland, United Kingdom, Germany, the Netherlands, and Denmark (Gillard, Thiébaut, Deleu, & Leroy, 2017). In this context, the dispersal in terrestrial habitat of *L. grandiflora* could also concern in the future these Europeans countries.

## CONFLICT OF INTEREST

None declared.

## AUTHOR CONTRIBUTIONS

D. Barloy coordinated all this study. All authors contributed to collecting data. Analysis of data and the first draft of the manuscript were carried out by K. Billet, J. Genitoni, D. Renault, and D. Barloy. All authors read and approved the final manuscript.
