# Supplementary material for: Aquatic and terrestrial morphotypes of the aquatic invasive plant, Ludwigia grandiflora, show distinct morphological and metabolomic responses
Source: Ecol Evol. 2018 Feb 4;8(5):2568–79. doi: 10.1002/ece3.3848 (PMC5838032; doi:10.1002/ece3.3848)
Supplement: Supplementary file 1 [file ECE3-8-2568-s001.pdf]

Aquatic and terrestrial morphotypes of the aquatic invasive plant, *Ludwigia grandiflora* show distinct morphological and metabolomic responses.

Kevin Billet[1], Julien Genitoni[1], Michel Bozec[1], David Renault[2,3], Dominique Barloy[1]*

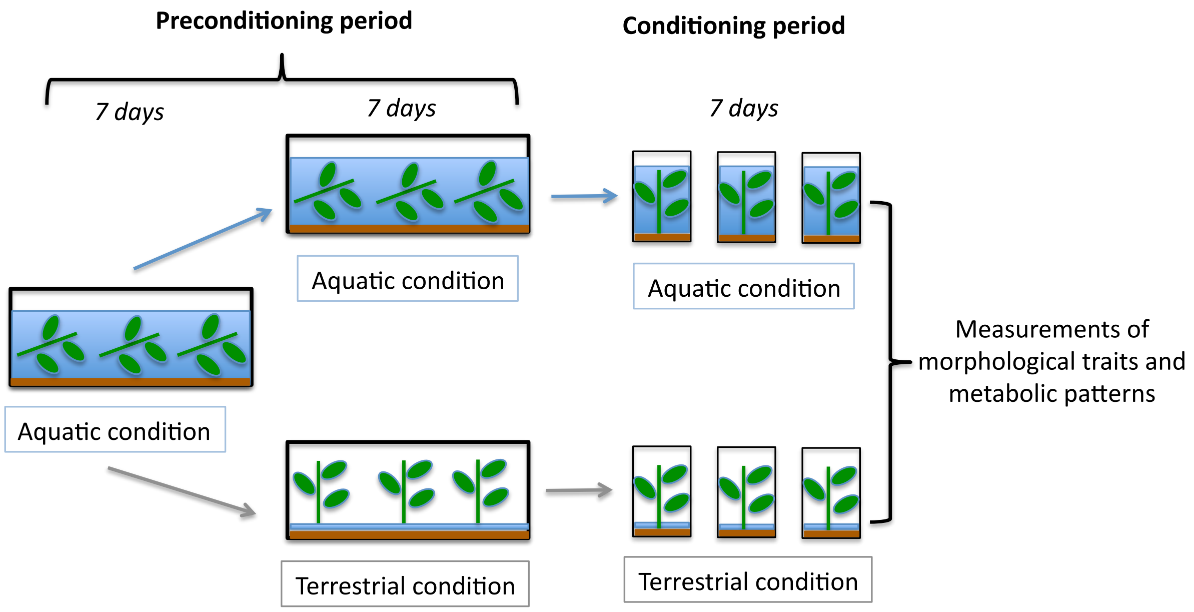

Figure S1: Experimental design of preconditioning and conditioning periods

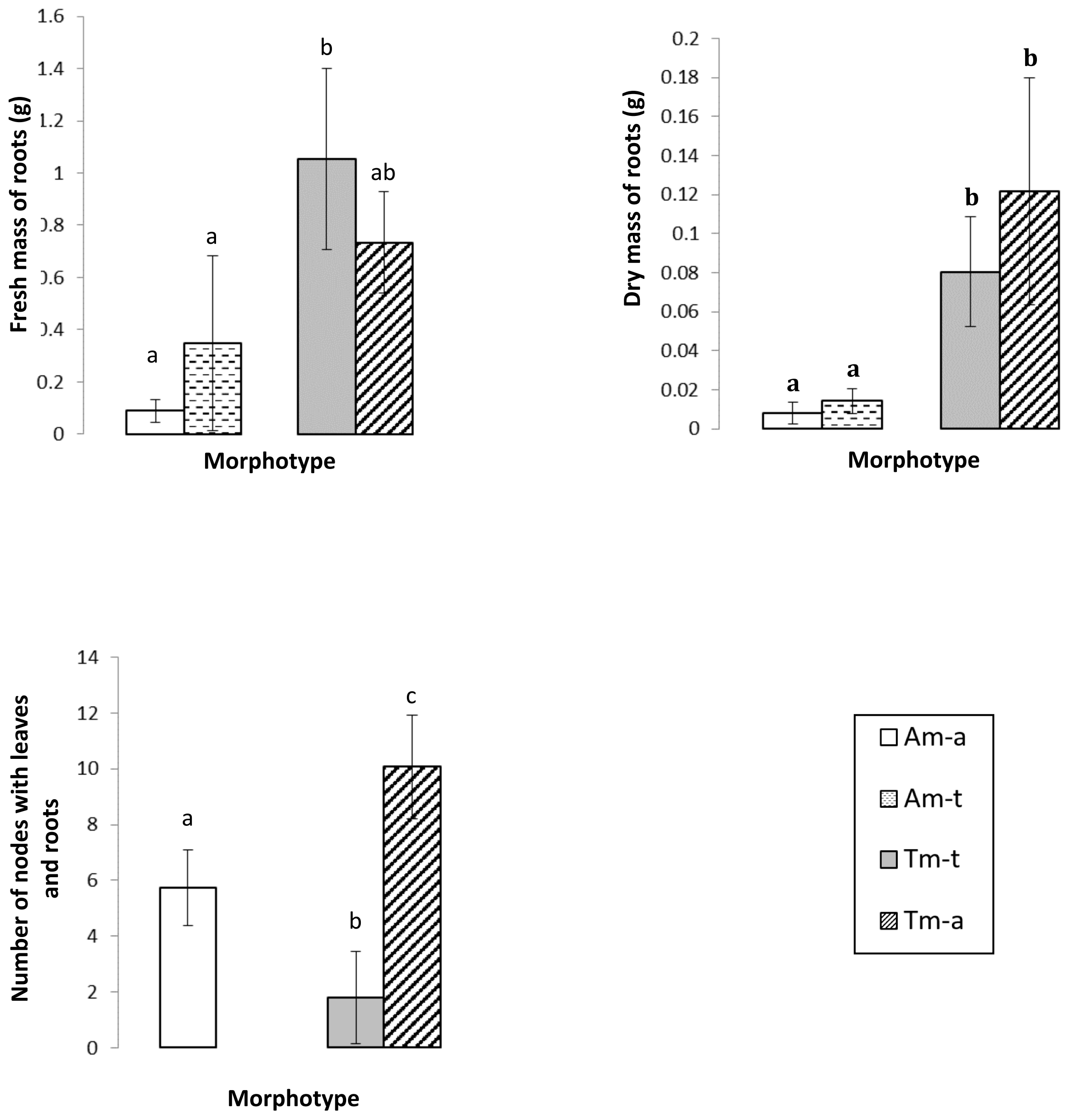

Figure S2: Variation of morphological traits of *L. grandiflora* in aquatic or terrestrial conditions

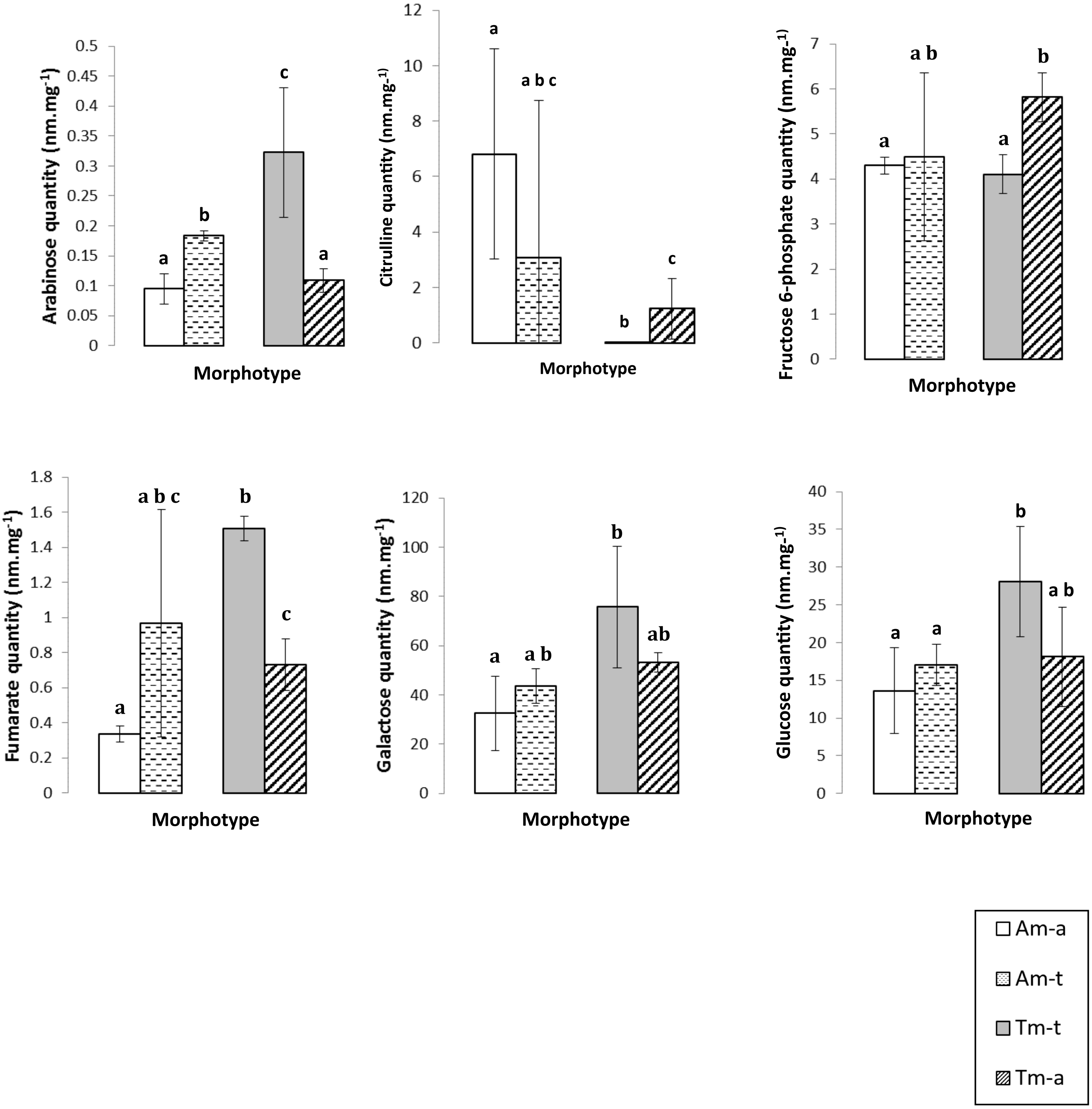

Figure S3: Changes in different metabolites involved in shoots L. *grandiflora* responses to aquatic or terrestrial conditions. Am-a and Am-t = aquatic morphotype in aquatic or terrestrial conditions; Tm-a and Tm-t = terrestrial morphotype in aquatic or terrestrial conditions, respectively. Same letter indicates non-different values (α=0.05).

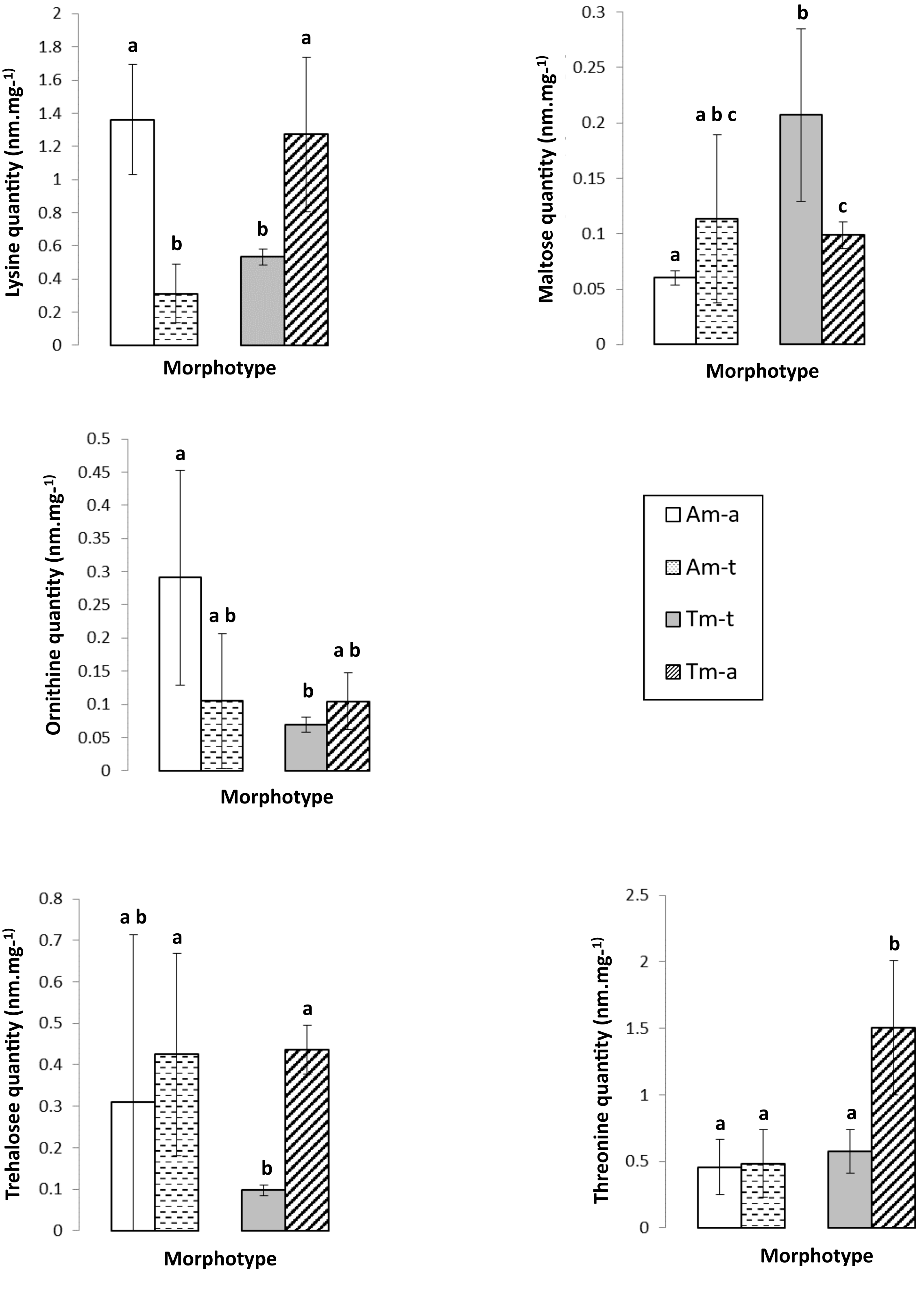

Figure S3: continued

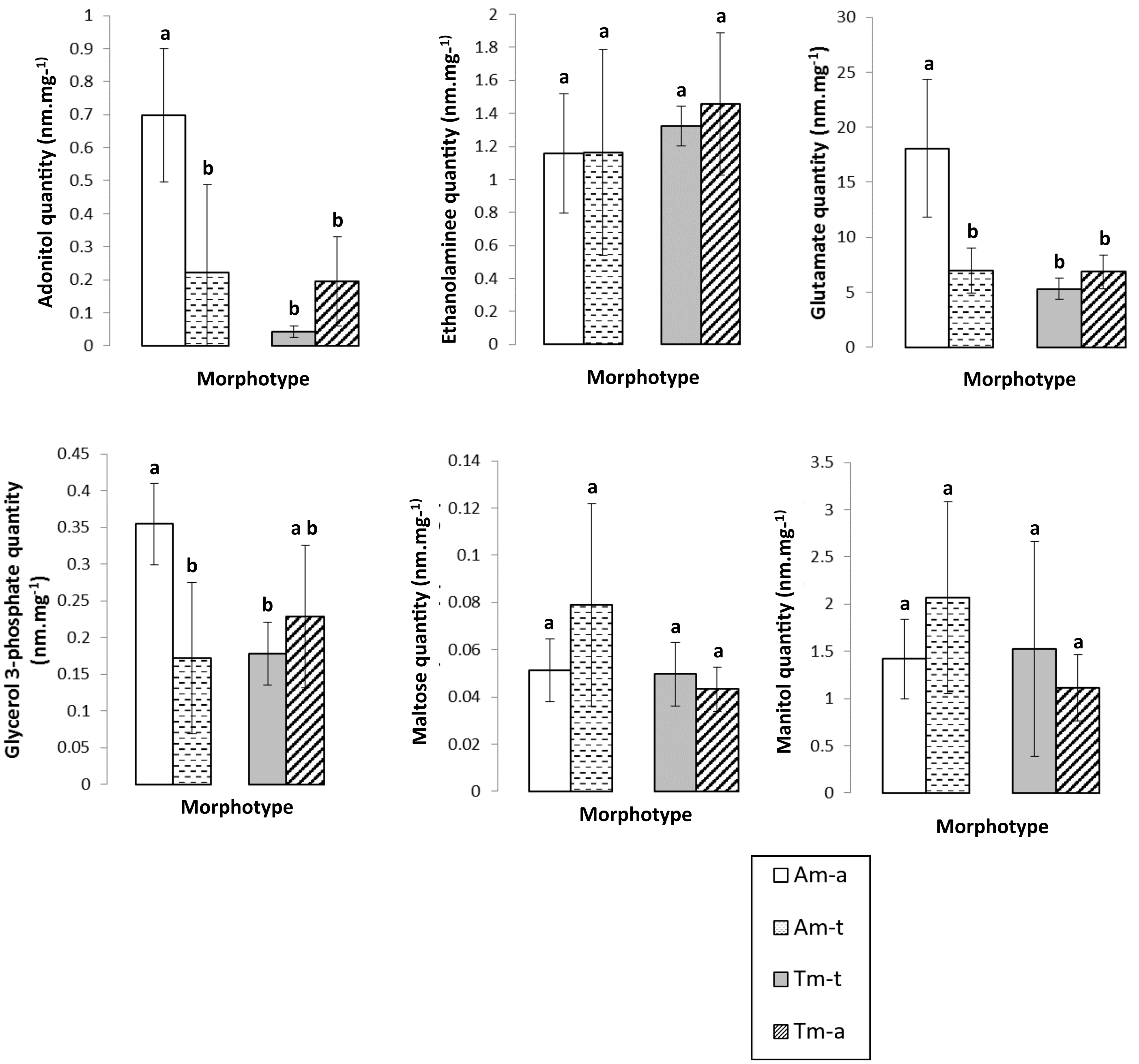

Figure S4: Changes in different metabolites involved in roots *L. grandiflora* responses to aquatic or terrestrial conditions. Am-a and Am-t = aquatic morphotype in aquatic or terrestrial conditions; Tm-a and Tm-t = terrestrial morphotype in aquatic or terrestrial conditions, respectively. Same letter indicates non-different values (α=0.05).

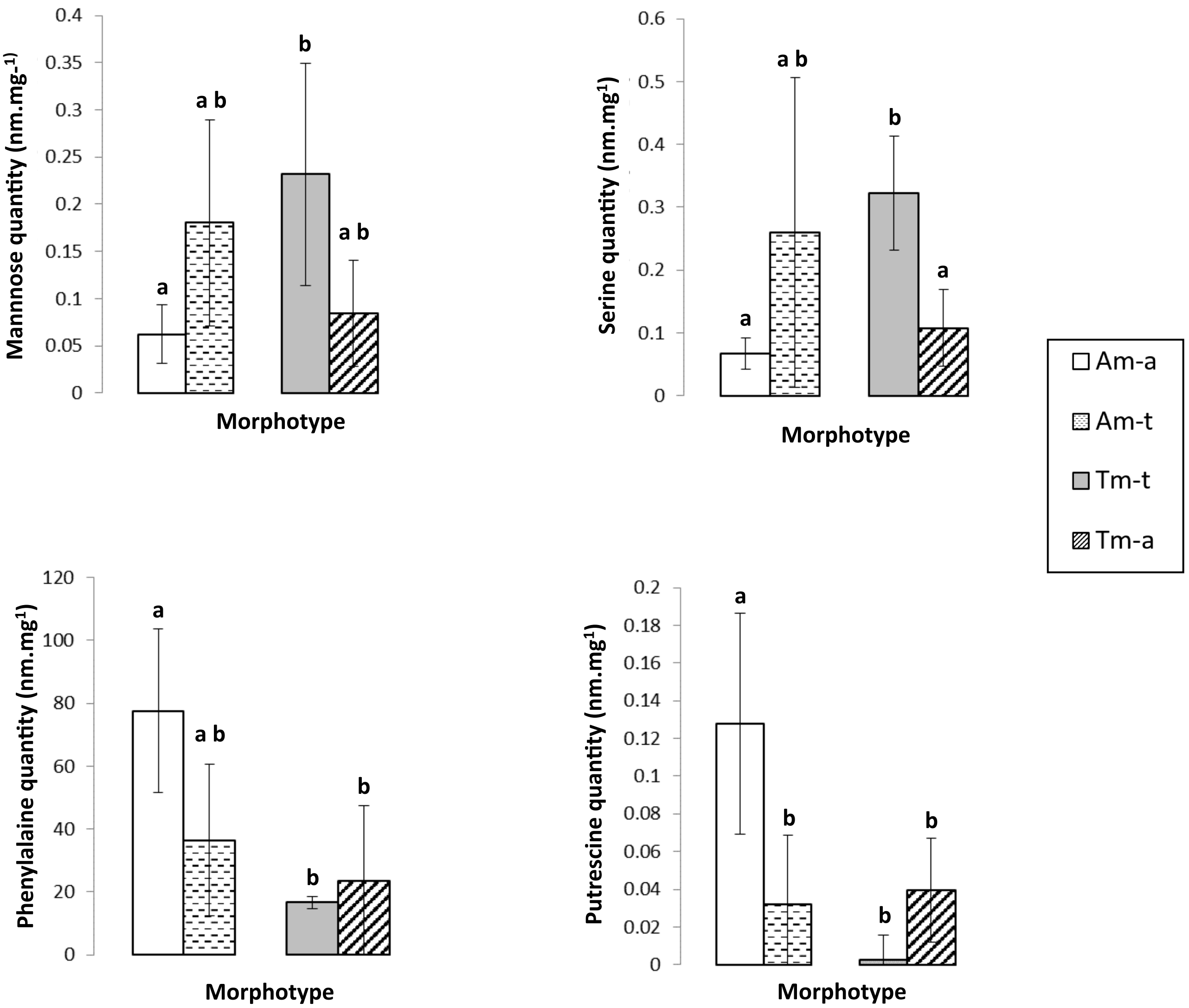

Figure S4 : continued

Table S1: List of Log and Cos transformed data

Morphological trait	Metabolites	
	**Tige**	**Racine**
LP	Alaline	Clucose
NbB	Aspartate	Galactose
FMS	F6P	Glutamate
DMS	Glycerate	Glycerol 3P
FMR	Isoleucine	Mannitol
DMR	Leucine	Mannose
Rr	Ornithrine	Serine
rr	Threonine	
